# Multisector Partnerships in Population Health Improvement

**Published:** 2010-10-15

**Authors:** Julie Woulfe, Thomas R. Oliver, Kirstin Q. Siemering, Susan J. Zahner

**Affiliations:** University of Wisconsin Population Health Institute, Madison, Wisconsin; Department of Population Health Sciences, University of Wisconsin School of Medicine and Public Health; University of Wisconsin Population Health Institute, Madison, Wisconsin; University of Wisconsin-Madison, School of Nursing, University of Wisconsin Population Health Institute, Madison, Wisconsin

## Abstract

Many new initiatives for population health improvement feature partnerships of leaders and organizations across multiple sectors of society. The purpose of this article is to review 1) the rationale for such partnerships as an important, if not essential, tool for population health improvement; 2) key organizational and contextual factors that appear to be associated with effective multisector partnerships; and 3) the limited evidence regarding the effect of such partnerships on population health outcomes. We conclude that systems thinking — accounting for the collective effect of many actors and actions — is essential to organizing and sustaining efforts to improve population health, and to evaluating them. More research is needed to understand how and why multisector partnerships are formed and sustained and the conditions under which multisector partnerships are necessary or more effective than other strategies for population health improvement. Research on and evaluation of multisector partnerships also need to incorporate more standard measures of partnership contexts, characteristics, and strategies and adopt longitudinal and prospective designs to accelerate social learning in this area. Finally, studies of multisector partnerships must be alert to the value of such initiatives to individuals and communities apart from any direct and measurable impact on population health.

## Introduction

In response to the call of the Institute of Medicine for multisector partnerships (1), many new initiatives for population health improvement feature partnerships of leaders and organizations across multiple sectors of society. These partnerships typically include representatives and resources from various substantive issue areas — for example, education, economic development, transportation, agriculture, and health — and span the business, nonprofit, and governmental sectors. The purpose of this article is to review 1) the rationale for such partnerships as a tool for population health improvement, 2) key organizational and contextual factors that appear to be associated with effective multisector partnerships, and 3) the limited evidence regarding the effect of such partnerships on population health outcomes.

## The Case for Multisector Partnerships

During the past 3 decades, efforts to improve population-wide health outcomes have moved toward community organizing and collaboration. Community organizing refers to the unit of analysis and action, shifting the focus from individuals to systems, rules, social norms, or laws to affect health behaviors and outcomes (2). This ecologic approach recognizes the connection between health and social institutions, surroundings, and social relationships (3).

Collaboration refers to the process of system change, shifting the focus from the responsibilities and effectiveness of individual institutions to their relationships and collective effect on population health. In particular, efforts have increased to involve many sectors of a community in pursuit of better health outcomes and the economic and social benefits thought to be associated with such outcomes. The rationale behind multisector partnerships is that, because no single organization or sector has full control over the determinants of population health, effective solutions require interorganizational coordination and collaboration (4). By pooling resources, talents, and strategies from a broad range of actors, each of these sectors can more effectively carry out its responsibilities as they affect population health (2). Researchers have advanced similar theories of collaboration to improve the effectiveness of initiatives on related issues such as poverty and community development.

Researchers have conceptualized partnerships for health improvement differently. Three dominant models of partnerships for health improvement have been described (4). In the first, public health agencies are primarily responsible for promoting activities and services that affect the health of the community. Their partnerships with other organizations exist primarily to extend the reach and capacity of governmental public health. In the second, many organizations play some role in promoting public health and so must be involved in health improvement. However, the focus remains primarily on the delivery of public health services. The third model focuses on the system of actors and actions that promote or threaten population health and includes activities in all sectors of community life (eg, education, business) (4). This last model, the most ecologic of the 3, has received increasing attention. However, the evidence to date suggests that these large-scale community health promotion projects have changed population health behaviors and outcomes only moderately (5).

In response to the mixed results of approaches based on the third model, some argue it is necessary to reconceptualize partnerships for health improvement (6). According to this argument, even the broadest partnerships have not shifted from an individual intervention paradigm to a true systems paradigm. Systems thinking focuses on the collective influence of a broad range of actors. It recognizes communities as networks of dynamic, nested relationships among individuals and organizations. These constantly evolving complex adaptive systems comprise diverse agents operating in various subsystems and suprasystems without centralized control (7). Although most partnerships adopt interventions targeting multiple levels within a system, they may fail to recognize the full scope and complexity of the system and miss opportunities to improve population health. Hawe and colleagues (6) argue that unique problems are associated with scaling up partnerships from the organizational level to the community level. They suggest that these partnerships learn from ecologic-systems perspectives that examine linkages, relationships, feedback loops, and interactions among systems. From this approach, multisector partnerships can be conceptualized as events within systems that either leave a lasting footprint or wash out, depending on how well the dynamic properties of the system are harnessed. The success of a partnership depends on activity settings, the social networks that connect people and settings, and time (6).

Recent work on social networking approaches to collaboration examines the importance of looking at the effect of a particular intervention rather than measuring the changes in a system over time. In network approaches, leaders focus not only on management challenges and opportunities at an organizational level but also on how to mobilize resources more broadly for the greatest social impact (8).

Drawing from these approaches, a fourth conceptualization of multisector partnership seems to emerge. This model focuses not only on the relationships among organizations in the partnership but also on the partnership's relationship to the context of the place it is trying to change. In some ways, this model is a continuation of the focus on neighborhood-based and community initiatives. However, it adds a new emphasis on considering the characteristics of context, including the timing of the intervention and past events, particularly earlier interventions that may have created networks. From this perspective, partnerships work to build capacity over time and consider the impact on the context itself as the primary outcome.

## Key Factors in the Effectiveness of Multisector Partnerships

Extensive research has identified the qualities perceived as contributing to strong multisector partnerships in health and other issue areas. This section summarizes some of the lessons learned about the most important dimensions of partnerships.

### Partnership resources

Partnership resources include the money, skills and expertise, information, and connections that a partnership has to draw on (9). Although resources alone do not ensure the success of partnerships, how partnerships are funded and supported does influence their functioning (10). Some common themes are the necessity of sufficient resources, the sustainability of resources, and whether funding supports the partnership's original mission and vision (8,9,11). In addition to sustainable funding, the flexibility of funding is important to long-term success (12). Coalitions may need access to information and support in the form of ongoing technical assistance (10), which enables the partnership to evaluate and change its efforts.

### Common vision for partnership

Multisector partnerships bring together groups with disparate interests and roles. One of the most universally recognized needs is a common vision for the partnership's projects, goals, and outcomes (13).

Partnerships without clear goals that rely on broad agendas may become distracted by emerging crises and side issues. Another risk is to become so narrowly focused that the partnership ignores important community and contextual issues. A related concern is ownership of the vision for the project. Researchers emphasize that communities that are being served by the partnership must contribute to the vision for the project, creating a sense of ownership and empowerment (10,14).

### Leadership

Effective leadership is one of the most studied characteristics of effective partnerships (10,15-17). Leadership style can vary from collaborative leadership to a more hierarchical model. Whatever the style, however, effective leadership inspires commitment and action, helps the partnership to work toward inclusion, and works to sustain the vision and participation of the partnership's members (10,15).

Research demonstrates the importance of building leadership at many levels. Along with leaders who possess expertise and experience in the issue area, collaborations need sponsors who can provide resources to the enterprise and champions who possess the necessary process-oriented skills to keep the collaboration going. Champions are particularly important because a diverse organizational partnership may lack a clear-cut strategy that can be centrally developed and easily enforced (18).

### Organizational structure

The effectiveness of partnerships depends on their organizational structure and capacity. As with leadership, no one form can serve all partnerships equally well. Effective partnerships appear to share several features, however, including clear structure, adequate staffing, sufficient core resources, and transparent decision-making processes (10,13,16).

A core test of organizational structure and process is the ability of a partnership to deal with conflict. In multisector collaborations, conflict is common and emerges from the marriage of different organizational cultures with varied views about planning, strategies, and tactics. Collaborations that have continual trust-building activities are more likely to manage potential conflict. Conflicts exist not only at an individual level but also at the systemic level. Consequently, collaborations are more likely to succeed when they build in resources and tactics for dealing with power imbalances (18). To achieve a broad consensus of how to proceed, the partnership should develop norms, rules, and processes based on the input of all members of the partnership. The planning must also involve the broader network of affected parties and attend to the  stakeholders (18).

### Membership

Selection of the right partners is necessary for success. Partnerships aimed at community health improvement should include a broad array of partner organization types (11). Membership diversity refers to members' social identity (ie, racial, ethnic, or cultural identity) and how well they represent the community the partnership serves (16). Building a culturally diverse membership increases the likelihood that the interventions will be culturally appropriate and strengthens the community's investment in the partnership. Attracting broad membership and community investment requires partnerships to demonstrate how their issues relate to the broader concerns of the partners and the community as a whole (13).

There are potential risks, however, in forming new collaborations. Recruitment of members presents a tradeoff between representativeness and effectiveness. Up to a point, expanding representation can increase legitimacy and attract more resources for an initiative. But coalition size and diversity may make it harder to reach decisions and develop and implement new programs (T. R. Oliver and J. Gerson, unpublished report to The California Endowment, October 2006).

Although newly constituted partnerships may have the advantage of not being obligated to any particular community group, they may lack credibility and power. Partnerships must therefore strategically align themselves with established groups (12). Bryson et al (18) found that cross-sector collaborations were more likely to succeed when 1 or more linking mechanisms (ie, existing networks, powerful sponsors) were already in place. Thus, building from existing relationships may be more effective than forging completely new ones (18). Research on which members are most valued by partnerships indicates that the most valuable member has a well-connected presence in the community, can devote resources to the collaboration, and actively participates (19).

Forty coalition leaders named commitment to the cause as the main element of coalition success. Additional factors named were commitment to coalition unity, breadth of representation, continuing contribution of resources, and previous history of working relationships (17).

### Quality of relationship

In addition to the desired structural characteristics of partnerships, the quality of the relationship distinguishes effective partnerships from ineffective ones. This sense of collaboration or group cohesion is complex and difficult to operationalize. Nonetheless, strong collaborative working relationships are often credited with allowing multisector partnerships to provide integrated service delivery (15,16). Good communication among partners, transparency in decision making, and accessible, jargon-free language better enable partners to participate effectively. Communication and ongoing feedback enable the partnership to grow and evolve. Effective partnerships have been successful in establishing a sense of mutual trust, respect, and commitment (13). Overall, effective coalitions and partnerships bond individuals in addressing a concern together, creating a sense of community and connection (10).

### External and contextual factors

The influence of community characteristics on the success of collaborations is a subject of growing interest. Some communities may have more readiness or be more conducive to the work of the partnership (9,10). Feinberg and colleagues (20) examined the relationship between 3 dimensions of community coalition readiness and the perceived effectiveness of the coalition. In a study that evaluated leadership readiness, community readiness, and strength of community ties, they found that community readiness is positively related to the perceived efficacy of coalitions (20). A community's readiness may be affected by capacity built through prior partnerships, the presence of competition between and within sectors, and the degree to which a community is already saturated with similar partnerships (10).

Communities each come with their own public and organizational policy barriers to partnerships. Financial barriers may include short-term or limited external funding, lack of funding for administration and management, and categorical program requirements. Other barriers may include performance standards or current benefit requirements that discourage key leaders or organizations from participating (9).

Although external factors affect the success of collaborations, the research on community coalitions suggests that the collaboration's response to those factors is more important to the development of the collaboration. Members of community coalitions routinely name  political, economic, and community conditions as important in coalition development. However, they identify additional factors as more important, such as choosing a relevant issue, having the right timing, and choosing an appropriate social target (17).

## Evidence of the Effectiveness of Partnerships

Despite a common belief that multisector collaboration can improve population health, researchers seldom study the effect of such collaboration on population health outcomes. Evaluating the effect of multisector partnerships on population health outcomes is difficult. Some of the most-cited challenges are the short study period of evaluations, limited use of evidence-based logic models and theories of action to guide interventions, the difficulty of measuring the degree of individual exposure to interventions, and multiple or broad population indicators (21).

Researchers fail to agree on what factors are most closely linked to improved population health outcomes. Often these factors have been drawn from a broad review of literature from multiple disciplines, each defining efficacy differently (14). Even researchers who agree that a particular quality of a coalition is important may disagree about how to measure that quality (16).

In a review of hundreds of collaborations, Roussos and Fawcett (21) could identify only 34 evaluations of partnerships working locally to address community health that had a study design or logic model to guide their work. Of the 34 partnerships, 10 presented improved population-level outcomes that might be attributed to collaboration activities. The review found stronger support for the ability of collaborations to change behavior and systems. Of the 34 studies of partnerships, 15 included measures of behavior change, 14 of which indicated some shift in behavior. All 34 studies reported some sort of systems change in the form of new programs developed, funds generated, or other measures (21).

Another literature review (16) yielded similar results. The authors searched major databases for studies on partnerships that targeted local geographic areas to improve population-level health outcomes, and defined and measured both coalition effectiveness and coalition-building factors. The review noted that across studies, researchers have defined and operationalized coalition-building factors and effectiveness differently. Studies had different definitions of coalition functioning, often failed to connect coalition-building factors to coalition effectiveness, and yielded mixed results (16). One study concluded that multisector partnerships and interventions continue to be driven primarily by ideology and action rather than sound scientific design and evaluation (22).

## Conclusions

Kreuter and Lezin (23) observe that justifications for collaborating to change health status and health systems fall into 2 major categories, conventional wisdom and evidence. Of the 2 justifications, conventional wisdom is vastly more common in the literature. The need for continued research and evaluation of broad-based initiatives to improve population health is clear, given the challenges of studying the influence of multisector partnerships in complex systems. Further research is needed to understand the circumstances in which formal multisector partnerships are likely to be formed, the extrinsic and intrinsic motivations of leaders and members, and how to increase the commitment of members through incentives and other means. In addition, further research is needed to identify whether and how multisector partnerships affect both the levels of population health and disparities within a population and to clarify what characteristics of partnerships and what contextual conditions are necessary for improved health outcomes. Finally, more research is needed to examine the comparative effectiveness of multisector partnerships and other strategies for improving population health, in particular, when the leadership and resources required to organize and maintain formal partnerships are *not* necessary to improve health outcomes or reduce health disparities.

General lessons are available: first, systems thinking is essential to organizing and sustaining efforts to improve population health, and to assessing their impact. The outcomes of partnership approaches depend on the social, economic, and political context of the community in which partnerships are formed and operate. Only by studying the varying contexts can researchers discern whether any form of partnership is sufficient for population health improvement.

Second, characteristics of partnerships — goals, sponsorship, membership, resources, leadership — do appear to matter, but this has been established primarily through studies based on perceptions of participants rather than objective measures of outcomes. Therefore, more research is needed on multisector partnership outcomes using longitudinal and prospective designs that include measurement of activities, social network development, and types of organizations involved and resources engaged. To aid this area of inquiry, better and more widely adopted measures of structure, process, and outcomes are needed to link partnership formation to community-wide impact. One step toward building a stronger evidence base of what works would be the adoption of common models or frameworks for defining different forms of public health partnerships — for example, the typology offered by Mays (4). Standard models, as well as more standard measures of partnership contexts, characteristics, and strategies, would improve the generalizability and replicability of research and accelerate learning.

Third, multisector partnerships almost certainly offer some value to individuals and communities apart from any direct and measurable effect on population health. The shared effort and communication that result from a health initiative may highlight problems, shift resources, or raise expectations for participation and performance in other areas of community life. Studies of multisector health partnerships should be alert to such catalytic changes and spillover effects as researchers pursue a clearer view of the connections between partnerships and population health improvement.

## References

[1] Institute of Medicine, National Academy of Sciences (2003). The future of public health in the 21st century.

[2] Butterfoss FD (2007). Coalitions and partnerships in community health.

[3] Anderson LM, Scrimshaw SC, Fullilove MT, Fielding JE, Task Force on Community Preventive Services (2003). The Community Guide's model for linking the social environment to health. Am J Prev Med.

[4] Mays GP (2010). Improving public health system performance through multiorganizational partnerships. Prev Chronic Dis.

[5] Merzel C, D'Afflitti J (2003). Reconsidering community-based health promotion: promise, performance, and potential. Am J Public Health.

[6] Hawe P, Shiell A, Riley T (2009). Theorising interventions as events in systems. Am J Community Psychol.

[7] Keshavarz N, Nutbeam D, Rowling L, Khavarpour F (2010). Schools as social complex adaptive systems: a new way to understand the challenges to introducing the health promoting schools concept. Soc Sci Med.

[8] Wei-Skillern J (2010). Networks as a type of social entrepreneurship to advance population health. Prev Chronic Dis.

[9] Lasker RD, Weiss ES, Miller R (2001). Partnership synergy: a practical framework for studying and strengthening the collaborative advantage. Milbank Q.

[10] Wolff T (2001). A practitioner's guide to successful coalitions. Am J Community Psychol.

[11] Zahner SJ (2005). Local public health system partnerships. Public Health Rep.

[12] Stagner MW, Duran MA (1997). Comprehensive community initiatives: principles, practice, and lessons learned. Future Child.

[13] Green L, Daniel M, Novick L (2001). Partnerships and coalitions for community-based research. Public Health Rep.

[14] Mayer JP, Sweid R, Dabney S, Brownson C, Goodman RM, Brownson RC (1998). Practices of successful community coalitions: a multiple case study. Am J Health Behav.

[15] Hayes CE, Hayes SP, DeVille JO, Mulhall PF (2000). Capacity for effectiveness: the relationship between coalition structure and community impact. Eval Program Plann.

[16] Zakocs RC, Edwards EM (2006). What explains community coalition effectiveness? A review of the literature. Am J Prev Med.

[17] Mizrahi T, Rosenthal BB (2001). Complexities of coalition building: leaders' successes, strategies, struggles, and solutions. Soc Work.

[18] Bryson JM, Crosby BC, Stone MM (2006). The design and implementation of cross-sector collaborations: propositions from the literature. Public Adm Rev.

[19] Varda DM, Chandra A, Stern SA, Lurie N (2008). Core dimensions of connectivity in public health collaboratives. J Public Health Manag Pract.

[20] Feinberg ME, Greenberg MT, Osgood DW (2004). Readiness, functioning, and perceived effectiveness in community prevention coalitions: a study of communities that care. Am J Community Psychol.

[21] Roussos ST, Fawcett SB (2000). A review of collaborative partnerships as a strategy for improving community health. Annu Rev Public Health.

[22] O'Neill M, Lemieus V, Groleau G, Frotin J, Lamarche P (1997). Coalition theory as a framework for understanding and implementing intersectoral health-related interventions. Health Promot Int.

[23] Kreuter MW, Lezin NL (1998). Are consortia/collaboratives effective in changing health status and health systems? A critical review of the literature.

